# Pituitary Gonadotropins, Prolactin and Growth Hormone Differentially Regulate AQP1 Expression in the Porcine Ovarian Follicular Cells

**DOI:** 10.3390/ijms19010005

**Published:** 2017-12-21

**Authors:** Mariusz T. Skowronski, Patrycja Mlotkowska, Damian Tanski, Ewa Lepiarczyk, Michal K. Oklinski, Soren Nielsen, Agnieszka Skowronska

**Affiliations:** 1 Department of Animal Physiology, University of Warmia and Mazury in Olsztyn, 10-752 Olsztyn, Poland; patrycja.mlotkowska@wp.pl (P.M.); damian.tanski@uwm.edu.pl (D.T.); 2 Department of Human Physiology, University of Warmia and Mazury in Olsztyn, 10-752 Olsztyn, Poland; ewa.lepiarczyk@uwm.edu.pl; 3 Department of Health Science and Technology, Aalborg University, 9220 Aalborg, Denmark; mko@hst.aau.dk (M.K.O.); sn@hst.aau.dk (S.N.)

**Keywords:** pig, ovarian follicles, pituitary hormones, aquaporin 1, in vitro study

## Abstract

The present in vitro study analyzed whether the hormones that affect the ovarian follicular steroidogenesis process also participate in the regulation of AQP1 mRNA and protein expression. Granulosa (Gc) and theca cells (Tc) of medium and large porcine ovarian follicles were exposed to follicle-stimulating hormone (FSH), luteinizing hormone (LH), prolactin (PRL) and growth hormone (GH) for 24 h in separated cells and co-cultures of these cells. Real-time PCR, Western blotting, immunofluorescence and volumetric analysis were then performed. Gonadotropins, PRL and GH had a stimulatory impact on AQP1 mRNA and protein expression in Gc and Tc of medium and large ovarian cells. Moreover, swelling assays, in response to a hypotonic environment, demonstrated the functional presence of AQPs in porcine Gc and Tc. Immunofluorescence analysis showed that AQP1 protein was mainly localized in the perinuclear region of the cytoplasm, endosomes and cell membranes of Gc and Tc from medium and large follicles. It seems possible that AQP1 present in Gc and Tc cells may be implicated not only in the regulation of water homeostasis required for follicle development but also in cell proliferation and migration.

## 1. Introduction

The hypothalamic-pituitary-ovary-uterus hormonal axis is essential for proper reproduction in pigs. Smitz and Cortvindt [1] drew attention to the importance of timing during reproductive processes of females; i.e., that the processes in the hypothalamus, pituitary, ovaries and uterus must occur in specific and precise temporal sequences. Growth and development of follicles take place through the selection, transformation of primordial follicle into primary, secondary and tertiary so-called pre-antral follicles. Next, the pre-antral follicles develop into small, medium and large follicles (the so-called “antral” follicles). In this follicular development, theca interna (Tc) and granulosa cells (Gc) play an important role. Luteinising hormone (LH) receptors are formed in Tc, and follicle-stimulating hormone (FSH) receptors in Gc, and further follicular growth and development are under the control of gonadotrophic hormones [2,3,4,5]. Studies on isolated Gc and Tc have provided evidence for their cooperation in this process [6,7]. During follicular growth, the ovum becomes mature when the level of estrogens inside the follicle is high and it is important in the mechanism of preovulatory LH surge in the pig [8,9]. Studies confirm that FSH is important because it raises the number of medium and large follicles and LH is required for further growth of these follicles for preovulatory use [10,11]. Formation of the antrum after stimulation with gonadotropins is a rapid process of follicle growth level of about 50× higher than during the pre-antral phase. This dramatic increase in follicle size forces rapid and massive water transport [12]. However, FSH and LH have a leading role in the growth regulation of ovarian follicles; additionally, prolactin (PRL) may be involved in gonadotrophic hormone action. Previous studies performed by Ciereszko et al. [13] showed that the PRL could be included in the hypothalamic-pituitary-ovary axis regulation during the pig cycle. The inhibitory effect of PRL on the production of estrogens has been shown to stimulate the production of progesterone by follicular cells. Prolactin promoted the synthesis of progesterone in Gc isolated from large follicles and inhibited progesterone in small follicles. A stimulatory effect of PRL on progesterone production by Tc and luteal cells was also found. The presence of PRL receptors was confirmed in most tissues, including ovaries [14,15,16]. It can be concluded, therefore, that PRL itself is important in the growth and differentiation of follicles. Other studies have shown that growth hormone (GH) also stimulates steroidogenesis in Gc and Tc of the pig follicle [17].

Aquaporins (AQPs) are membrane proteins that facilitate water transport via the plasma membranes. AQP1 is permeable to water, ions and gases [18]. AQP1 is mainly expressed in the vessels [18] and other human and animal tissues including, for example, the kidney [18], salivary glands [19], gastrointestinal tract [20,21], spinal cord [22] as well as in many types of cancer [23]. To date, ten AQP isoforms (1, 2, 3, 4, 5, 6, 7, 8, 9 and 11) have been found in the female reproductive system in mammals. The specific localization of AQPs throughout the system provide indirect evidence of their role in uterine imbibition mechanism, ovum transport and oviductal fluid balance, follicle maturation, blastocyst formation and embryo implantation [24].

Studies of ovarian AQPs expression are not as numerous as those of the uterus or fetal membranes. Previous studies have shown that AQPs are involved in the formation of human and rodent follicular antrum [25,26] as well as in folliculogenesis in pigs [27]. McConnell et al. [28] found that rapid water flow into the rat follicle cavities is mainly via Gc transmembrane transmission with AQP7, AQP8 and AQP9 involvement. Recently, Starowicz et al. [29] discovered the presence of AQP5 in the rat pre-ovulatory ovarian follicles, indicating the potential role of this protein in the periovulatory period. In turn, Qu et al. [30] demonstrated *Aqp9* expression in granulosa cells of patients with polycystic ovary syndrome. In contrast, AQP3 was indicated in Gc and Tc of pre-ovulatory follicles in women and mouse oocytes [25,26].

A study of AQPs on the pig model showed expression of AQP1, 5 and 9 in the ovary [27,31]. AQP1 was found in the capillary endothelium and AQP5 and AQP9 in Gc of primordial and developmental follicles. Because of this specific localization of these AQPs, it can be assumed that in porcine ovarian follicles, the expression of AQP1 in Tc would be of importance to transport water into the interstitium underlying the basal lamina and that water would pass through this membrane passively to be transported by AQP5 and 9 in Gc into the antrum. This study provides a very good basis for further in-depth research into the physiological role of AQPs in the pig ovaries. Therefore, the hypothesis of the present study assumed that hormones FSH, LH, PRL and GH that affect the ovarian follicular steroidogenesis, participate in regulation of AQP1 mRNA and protein expression.

## 2. Results

### 2.1. The Effects of FSH, LH, PRL and GH on Aqp1 mRNA Expression in the Gc and Tc Cells from the Medium and Large Follicles

**Figure 1:**
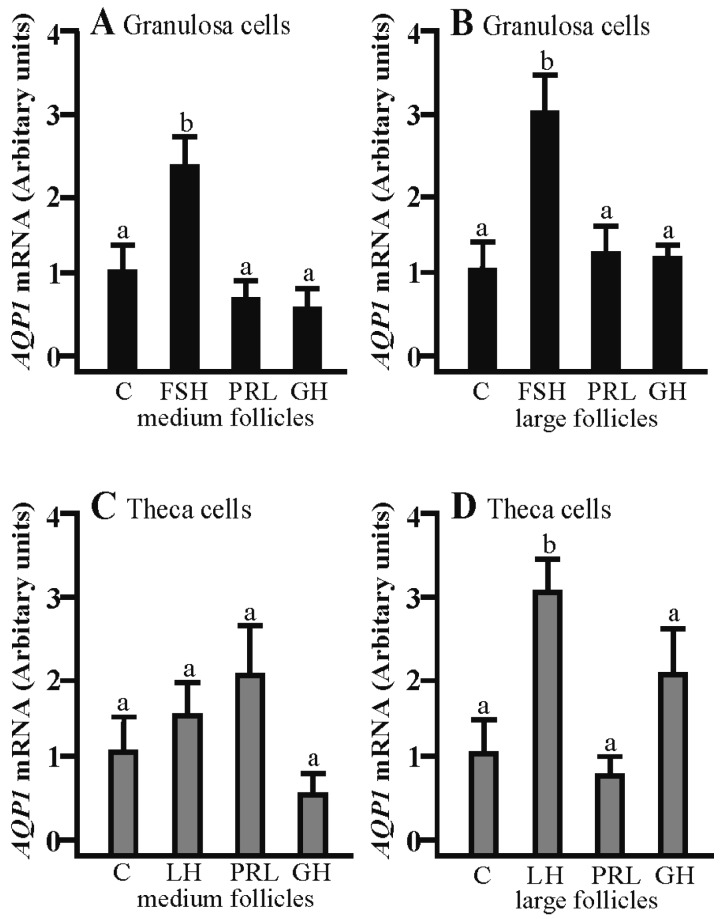
Effect of follicle-stimulating hormone (FSH), luteinizing hormone (LH), prolactin (PRL) and growth hormone (GH) on *Aqp1* expression in medium and large follicles. (**A**,**B**) in the granulosa cells and theca cells (**C**,**D**). FSH, follicle-stimulating hormone; LH, luteinizing hormone; PRL, prolactin; GH, growth hormone. The mRNA expression level of *Aqp1* was determined by real-time PCR and normalized to the expression of *GAPDH* and *PPIA*. Values are expressed as means ± S.E.M from five separate experiments, each performed in triplicates (*p* < 0.05 compared with controls). Statistically significant differences between treatments are indicated by different letters (a, b). C: control.

*Aqp1* mRNA expression was significantly increased in the Gc cells isolated from middle and large follicles after a 24 h culture with FSH as compared to the control (*p* < 0.05; Figure 1A,B). Other treatments did not affect *Aqp1* mRNA in the examined cells. LH increased *Aqp1* mRNA expression in the Tc cells obtained from large follicles (*p* < 0.05; Figure 1D). Other treatments did not affect *Aqp1* mRNA expression in Tc at the examined time point in comparison to the control group (Figure 1C).

### 2.2. The Effects of FSH, LH, PRL and GH on AQP1 Protein Expression in the Gc and Tc Cells from Medium and Large Follicles

**Figure 2:**
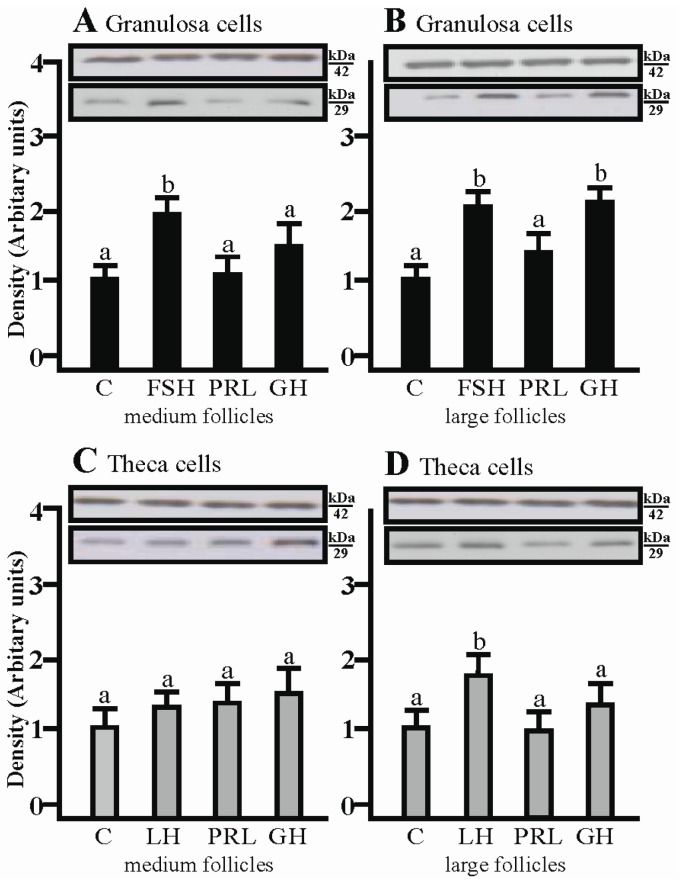
Effect of FSH, LH, PRL and GH on AQP1 protein expression in medium and large follicles. (**A**,**B**) in the granulosa cells and theca cells (**C**,**D**). FSH, follicle-stimulating hormone; LH, luteinizing hormone; PRL, prolactin; GH, growth hormone. Values are expressed as means ± S.E.M from five separate experiments, each performed in triplicates (*p* < 0.05 compared with controls). AQP1 protein levels was performed (29 kDa) and normalized against β-actin (42 kDa). Values are expressed as means ± S.E.M (*p* < 0.05, compared with controls). Statistically significant differences between treatments are indicated by different letters (a, b). C: control.

SDS-PAGE and Western blot analysis revealed that FSH increased AQP1 protein expression in porcine granulosa cells obtained from medium and large follicles (*p* < 0.05; Figure 2A,B) in comparison to the control. In addition, increased AQP1 expression was found in Gc cultured with GH for 24 h (*p* < 0.05; Figure 2B). As well as mRNA, it was found that AQP1 protein was detectable in Tc isolated from large porcine follicles cultured with LH (*p* < 0.05; Figure 2D). The tested hormones did not affect the Tc isolated from medium follicles.

### 2.3. The Effects of FSH, LH, PRL and GH on Aqp1 mRNA Expression in the Co-Culture of Gc and Tc Cells from Medium and Large Follicles

**Figure 3:**
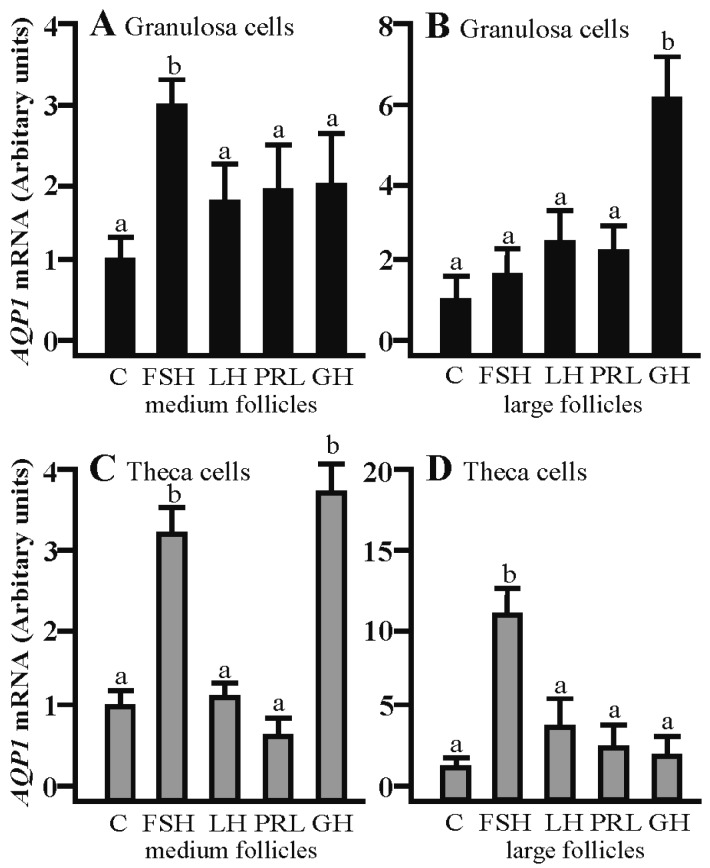
The expression of *Aqp1* mRNA in granulosa and theca cells co-cultured in the presence of FSH, LH, PRL, GH in medium and large follicles. (**A**,**B**) the granulosa cells and theca cells (**C**,**D**). FSH, follicle-stimulating hormone; LH, luteinizing hormone; PRL, prolactin; GH, growth hormone. Values are expressed as means ± S.E.M from five separate experiments, each performed in triplicates (*p* < 0.05 compared with controls). Statistically significant differences between treatments are indicated by different letters (a, b). C: control.

The co-culturing of Gc with theca cells from the medium follicles in the presence of FSH significantly increased *Aqp1* mRNA expression compared to the respective control (*p* < 0.05; Figure 3A). The addition of GH produced a significant increase in *Aqp1* mRNA expression in the Gc of large follicles (*p* < 0.05; Figure 3B). A stimulatory effect on *Aqp1* mRNA was observed in the Tc co-culture with granulosa cells in the presence of FSH and GH (*p* < 0.05; Figure 3C) in turn, in large follicles, this effect was only observed in the presence of FSH (*p* < 0.05; Figure 3D).

### 2.4. The Effects of FSH, LH, PRL and GH on AQP1 Protein Expression in the Co-Culture of Gc and Tc Cells from Medium and Large Follicles

**Figure 4:**
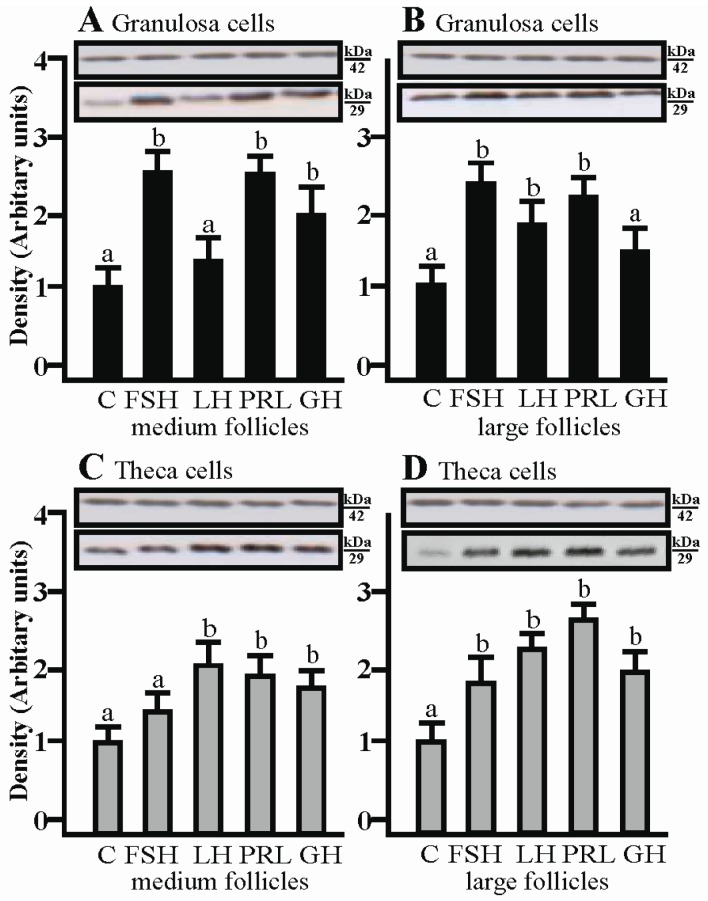
The expression of AQP1 protein in granulosa and theca cells co-cultured in the presence of FSH, LH, PRL, GH in medium and large follicles. (**A**,**B**) the granulosa cells and theca cells (**C**,**D**). FSH, follicle-stimulating hormone; LH, luteinizing hormone; PRL, prolactin; GH, growth hormone. Values are expressed as means ± S.E.M from five separate experiments, each performed in triplicates (*p* < 0.05 compared with controls). AQP1 protein levels was performed (29 kDa) and normalized against β-actin (42 kDa). Statistically significant differences between treatments are indicated by different letters (a, b). C: control.

AQP1 protein expression significantly increased in the co-culture of Gc with Tc isolated from medium follicles after treatment with FSH, PRL and GH, respectively (*p* < 0.05; Figure 4A), whereas in Gc obtained from large follicles, a significant increase in AQP1 protein was observed after treatment with FSH, PRL and LH (*p* < 0.05; Figure 4B). The increased AQP1 protein expression in co-culture Tc from large follicles was comparable to Gc; additionally a significant increase of AQP1 protein was observed after treatment with GH (*p* < 0.05; Figure 4D). In the co-culture of Tc with Gc obtained from medium follicles, AQP1 protein expression significantly increased with LH, PRL and GH (*p* < 0.05; Figure 4C).

### 2.5. The Subcellular Expression of AQP1 Protein in Porcine Gc and Tc from the Medium and Large Follicles after In Vitro Treatment with FSH, LH, PRL and GH

**Figure 5:**
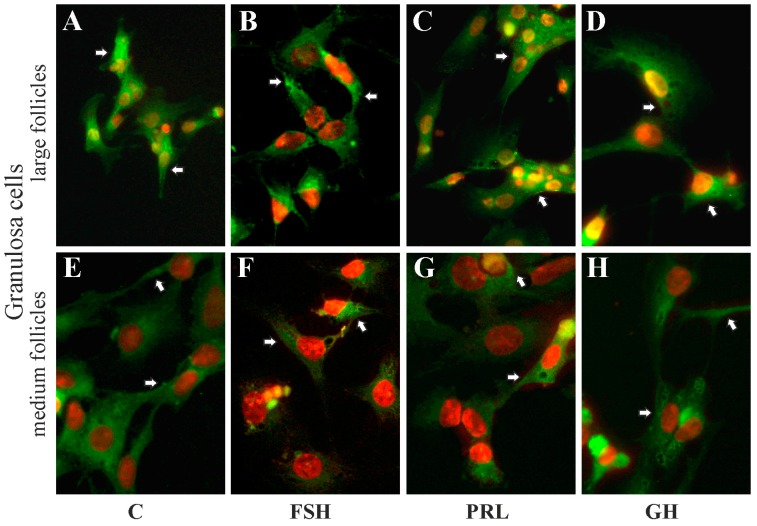
The influence of FSH, PRL and GH on subcellular distribution of AQP1 in granulosa cells of medium and large porcine ovarian follicles. Cells were exposed to FSH (**B**,**F**), PRL (**C**,**G**) and GH (**D**,**H**) for 24 h. The cells were fixed and incubated with anti-AQP1 antibody. Alexa-488 was used to visualize AQP1 (in green). Nuclei were stained with propidium iodide (in red). Arrows indicate localization of AQP1 in the cells. The data are representative of five separate experiments, each performed in duplicates. C: control (**A**,**E**); magnification of 600×.

**Figure 6:**
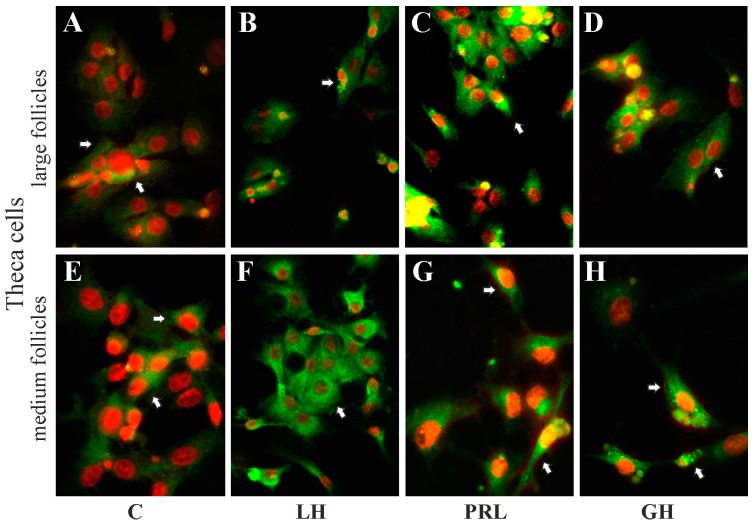
The influence of LH, PRL and GH on subcellular distribution of AQP1 in theca cells of medium and large porcine ovarian follicles. Cells were exposed to LH (**B**,**F**), PRL (**C**,**G**) and GH (**D**,**H**) for 24 h. The cells were fixed and incubated with anti-AQP1 antibody. Alexa-488 was used to visualize AQP1 (in green). Nuclei were stained with propidium iodide (in red). Arrows indicate localization of AQP1 in the cells. The data are representative of five separate experiments, each performed in duplicates. C: control (**A**,**E**); magnification of 600×.

Immunofluorescence analysis showed that AQP1 protein was mainly localized in the perinuclear region of the cytoplasm, endosomes and cell membranes of granulosa (Figure 5A–D) and theca (Figure 6A–D) cells from the large follicles as well as granulosa (Figure 5E–H) and theca (Figure 6E–H) cells from the medium follicles. There was no effect on subcellular distribution of AQP1 protein in these cells after 24 h treatment with FSH, LH, PRL and GH compared to respective controls (Figure 5 and Figure 6).

### 2.6. AQPs Are Functionally Expressed in Porcine Granulosa and thEca Cells

**Figure 7:**
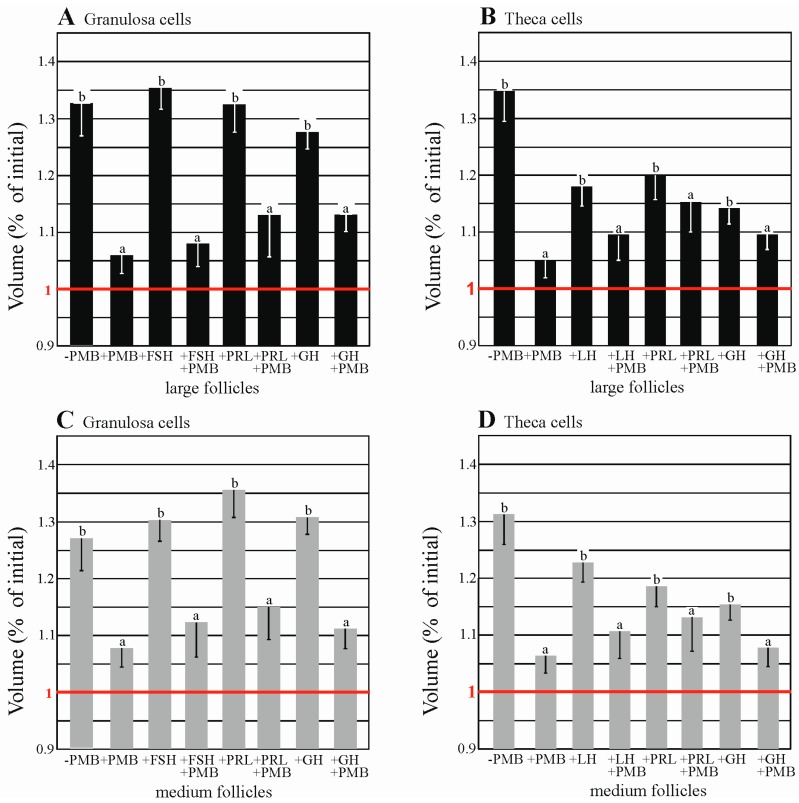
The effect of FSH, LH, PRL and GH on the swelling of the granulosa (**A**,**C**) and theca (**B**,**D**) cells of medium and large porcine ovarian follicles in hypotonic medium. In the assay, both groups of cells were randomized into four experimental groups and exposed to the examined factors for 24 h. The first group (+PMB) was the control group. The second group was treated with examined factors without PMB and the third group, after treatment with the studied factors, was incubated with the blocker (+PMB). The fourth group included cells treated without PMB (−PMB). All of the cells were washed three times with PBS and exposed to hypotonic medium for 30 s. Photographs were taken from 0 to 30 s at intervals of 3 s. An Olympus analysis platform (Olympus, Tokyo, Japan) was used to measure the volume of the cells. Data are mean ± S.E.M of five separated measurements of five separated experiments performed on different days. Statistically significant differences between treatments are indicated by different letters (a, b). 1 = initial volumes at the beginning of the experiments; line in red.

As shown in Figure 7, Tables S1 and S2, in the swelling assay, in the hypotonic solution the volume of Gc and Tc (−PMB) significantly increased (*p* < 0.05), in comparison to the volume of the cells in the presence of 50 µM HgCl_2_, AQPs blocker (+PMB). A significant increase in the volume of granulosa and theca cells from medium and large follicles treated with FSH, LH, PRL and GH was demonstrated when compared to the control group. Treatment with these hormones in the presence of PMB caused an increase in the cell volume which was not significant (*p* > 0.05). The data indicated that functional AQPs are expressed in the granulosa and theca cells of medium and large porcine ovarian follicles.

## 3. Discussion

The data support the proposal that the pituitary gonadotropins, prolactin and growth hormone considerably influenced AQP1 expression in the porcine ovarian follicular cells. In a previous study [31], the presence of AQP1 protein in the vascular endothelium of the entire porcine ovarian follicles during the estrous cycle and early pregnancy was demonstrated. Nonetheless, AQP1 protein expression was highest on days 18–20 of the cycle. Based on the above results, for the present in vitro studies, medium and large pre-ovarian follicles were selected and the granulosa and theca cells were separated from follicles and exposed to hormones (FSH, LH, PRL and GH) which affect the physiological functions of the ovarian follicle. The results of the present experiment demonstrate that AQP1 protein was localized in the cytoplasm (perinuclear region and in endosomes) and membranes of granulosa and theca cells of medium and large ovarian follicles, both in the control tissue and after treatment with FSH, LH, PRL and GH. Interestingly, a similar pattern (but of AQP5 expression) has recently been demonstrated in rat parotid acinar cells [32]. In the current study, the theca cells were deprived of blood vessels, although the AQP1 protein was expressed in these cells and, moreover, in granulosa cells. Thus, it may be assumed that AQP1 in granulosa and theca cell membranes is associated with the regulation of water transport, whereas in their cytoplasm it may be associated with the action of steroid hormones and participation in the growth of follicles. Previous studies reported an effect of steroid hormones on aquaporin expression in the female reproductive system [28,33,34]. Specifically, AQP2 and AQP5 are up-regulated by estrogen as they both contain estrogen sequences in the promoter regions, as shown in the rodent and human uterus [35,36]. Estrogens are the most biologically active steroid hormones during the estrous cycle in pigs. They stimulate follicular cell proliferation and, together with FSH, initiate the formation of LH receptors in granulosa cells, mainly in the mural, progesterone and androgen production in theca interna cells [37,38]. A thorough discussion regarding the influence of steroid hormones on aquaporins in the female reproductive system has been already presented in our previous papers [39,40,41]. In turn, very interesting results on transgenic mice deficient in AQP proteins showed different reproductive phenomena in both males and females [42,43,44,45].

Interestingly, the specific localizations of AQP1 in separated theca and granulosa suggest that AQP1 is not only responsible for high water permeability, but also for the migration and proliferation of ovarian cells. In general, migration is a fundamental property of cells that occurs in many physiological and pathological processes, including embryonic organogenesis, repair of damaged tissue after injury, inflammatory reaction, formation of new blood vessels and the spread of cancer [46]. Papadopoulos et al. [47] suggest that aquaporins have an effect on the selective transport of water through membranes in the cell, and fulfill at least two important functions in the migration process. They make it easier to change the shape of the cell and help to drive the cell forward. In turn, Saadoun et al. [48] indicate that AQP-dependent cell migration may be a general phenomenon but independent of AQP and cell types. Interestingly, the authors have shown that AQP1 is polarized to the frontal end of migratory cells, suggesting a significant role in the formation of the appendages at the leading edge of the cell. McCoy and Sontheiner [49] and Papadopoulos et al. [47] indicated the contribution of AQP1 and AQP4 in the migration of reactive astrocytes to the site of glial scars, which is a consequence of brain damage. Galán-Cobo et al. [50] confirmed the direct effect of AQP1 on cell proliferation. In turn, Yang et al. [51] suggested that AQP1 is involved in the differentiation and development of malignant ovarian tumor cells. In this context, it is tempting to assume that AQP1 participates in the process of migration and proliferation of porcine ovarian follicle cells in vitro.

Cell proliferation and growth are closely related to changes in cell volume [52]. Factors affecting cell volume also have implications for mechanisms that control cell proliferation [53]. To examine the presence of AQPs in granulosa and theca cells, a swelling assay was performed using an AQP blocker. Interestingly, the increase in volume of granulosa cells of medium and large follicles in hypotonic conditions after treatment with FSH, PRL and GH was higher compared to the theca cells of medium and large follicles under the same osmotic conditions with LH, PRL and GH. It is probable that granulosa cells have more AQP isoforms in the cell membranes than the theca cells. In recent studies [31], the presence of two isoforms (AQP5 and 9) has been demonstrated in granulosa cells and one (AQP1) in the theca cells. In an earlier study [27], immunohistochemical analysis was performed using nine anti-AQP antibodies (AQP1, 2, 3, 4, 5, 7, 8, 9 and 11) to investigate whether these proteins are expressed in pig ovarian cells. The analysis confirmed only the expression of AQP1, AQP5 and AQP9. Nevertheless, it must be stressed that it is not possible to exclude the presence of other examined AQP isoforms in these cells because the remaining antibodies used in the study may have been non-specific in this species. A study of cell volume changes is commonly used to evaluate AQP functions and is based on the fact that fluid transport occurs via the intact cell membrane under hypotonic conditions to achieve osmotic balance between the membrane [28]. As influenced by fluid infiltration from the hypotonic environment, the cell expands and this change can be observed with a contrast microscope [54]. The presence of three isoforms of AQPs (AQP7, 8 and 9) has been demonstrated in rat ovarian granulosa cells [28] and two isoforms (AQP3 and 7) in mouse oocytes [26,55]. McConnell et al. [28] showed the presence of AQP7 in granulosa cells. AQP7 is one of two isoforms (AQP4 and AQP7) insensitive to mercury compounds, suggesting that hypotonic swelling in the presence of mercury can, in fact, be the result of water transport through AQPs. In the present study, an increase in the volume of granulosa and theca cells induced by a hypotonic environment was inhibited by a PMB blocker, indicating that water transport via AQPs in these cells is physiologically significant. A volumetric experiment was performed for the first time in separated granulosa and theca cells of medium and large porcine ovarian follicles.

The present results have also revealed that the AQP1 protein can be regulated by FSH, LH, PRL and GH. In granulosa cells obtained from medium and large ovarian follicles, FSH significantly stimulated the expression of AQP1 mRNA and protein. However, in theca cells, only LH significantly increased the expression of *Aqp1* mRNA in large follicles. Expression of *Aqp1* mRNA in granulosa and theca cell co-cultures of medium follicles was significantly higher in response to FSH. Furthermore, the expression of *Aqp1* mRNA in the co-cultures of theca cells of medium and large ovarian follicles was significantly regulated by LH. The expression of the AQP1 protein was also significantly increased by LH in the theca cells of large follicles. AQP1 protein expression significantly increased in granulosa cells of medium and large follicles as well as in theca cells of large follicles after treatment with FSH in co-cultures of these cells. LH-treatment caused an increase in AQP1 protein expression in theca cells of medium and large follicles and in granulosa cells derived from large follicles. Thus, it may be assumed that gonadotropins exert their effect on the porcine follicles by influencing the mRNA and protein expression of AQP1. Thoroddsen et al. [25] showed high expression of *Aqp1*–*4* mRNA in granulosa and theca interna cells of women at precisely defined ovulation stages. The authors observed an increase in AQP1 protein expression immediately after follicular rupture, indicating that AQP1 is involved in the process of follicle transformation into corpus luteum. The growth of follicles and their differentiation and steroidogenic activity are controlled by many factors, of which FSH, LH, PRL and oxytocin are crucial [56]. Steroid and protein hormones are produced by the follicles, which, by auto- and paracrine pathways, affect follicular cell functions. In the present experiment, the observed disparity between the level of an mRNA transcript and that of its corresponding protein may result from differential stability of mRNAs and/or proteins as well as post-transcriptional regulation.

Growth factors, cytokines and interleukins are essential follicular regulators [11]. Many authors have emphasized that the growth of follicles and steroidogenesis is controlled by the interaction of insulin-like growth factors (IGF-S) and gonadotrophins [56]. Attention has also been paid to metabolic hormones; among others, GH affects the follicular function by the hypothalamic-pituitary axis or acting directly on the ovary. There is increasing evidence that GH and IGF-S play an important role in both follicular development and in their atrezia [57]. GH and locally produced IGF-I may modulate folliculogenesis [57]. Gregoraszczuk et al. [17] demonstrated the direct effects of GH on steroidogenesis in swine granulosa cells. In addition, Kolodziejczyk et al. [58] found that granulosa and theca cells produce IGF-I and showed the effect on proliferation of these cells. In the literature, there is no data on the influence of PRL and GH on the expression of aquaporins in the porcine ovarian cells. The research concerns only the influence of these hormones on AQP3 expression in Mozambique tilapia (*Oreochromis mossambicus*) gill epithelium [59]. The authors in this study devoted attention to the effect of osmoregulatory hormones such as PRL, GH and cortisol on AQP3 expression. They found an opposing action of PRL and cortisol on AQP3 expression at both mRNA and protein levels in marine and fresh water environments. The current study also demonstrated a stimulatory effect of PRL, however, only on AQP1 protein expression in the co-cultures of granulosa and theca cells of medium and large follicles. Growth hormone increased the expression of AQP1 protein in granulosa cells isolated from large follicles. Furthermore, the expression of *Aqp1* mRNA was significantly higher under the influence of GH in co-cultures of granulosa cells of large follicles as well as theca cells of medium follicles.

In conclusion, the present study, for the first time, has provided some novel insights into the regulation of AQP1 present in granulosa and theca cells of porcine ovarian follicles. It was demonstrated that gonadotropins and growth hormone increased AQP1 expression in granulosa and theca cells of medium and large follicles. Additionally, it was shown, in a co-culturing study, that prolactin had a stimulatory effect on AQP1 expression. It seems possible that AQP1 may also be implicated in the cell proliferation and migration.

## 4. Materials and Methods

### 4.1. Animals

All experiments were performed in accordance with the Animal Ethics Committee (AEC approval No. 66/2010 DTN, 15 June 2010, University of Warmia and Mazury in Olsztyn, Poland. Tissue samples were recovered from mature cross-bred gilts (Large White × Polish Landrace) aged 7–8 months (*n* = 20), with an average weight of 90–110 kg in a local slaughterhouse (Biskupiec, Poland). Ovaries were separated from all of the gilts and were then stored on ice and transported in cold-buffered physiological saline (PBS) supplemented with gentamycin and nystatin. The morphology of the ovaries were evaluated as describe previously [60]. The follicles were divided in two groups based on size: medium follicles (6–8 mm diameter) and large, pre-ovulatory follicles (9–12 mm).

### 4.2. Cell Cultures and Experimental Design

Granulosa cells (Gc) and theca interna cells (Tc) were subsequently prepared according to the technique described by Stokłosowa et al. [61] using a modification [62]. All stages of experiments were performed in sterile conditions. The total number of follicles (*n* = 10–12) for each group was used to obtain separated granulose and theca cells. Using a pair of fine forceps, theca interna/granulosa were separated from external layers of the follicular wall. Gc were scrubbed from the follicular wall with round-tipped ophthalmologic tweezers and rinsed off by intensive pippeting (10 s) and a supernatant containing granulosa cells was decanted. After isolation, Gc were rinsed in M199 medium with 5% BSA and centrifuged (180× *g*, 10 min, 20 °C). Then, the cell pellet was treated with a red blood cell lysing buffer as describe by [62] and the Gc were re-suspended in M199 supplemented with BSA 5% and antibiotics and counted in Burker’s chamber. Cell viability was determined by trypan blue dye exclusion and was always greater than 90%. The Tc was mechanically separated from the underlying theca externa cell layer. Tc were washed with PBS, and exposed to trypsinization with 6–7 mL, 0.25% trypsin in PBS for 10 min at 37 °C. The cells were filtered through a nylon mesh. Finally, the cells were centrifuged and re-suspended in M199/BSA and antibiotics.

Experiment 1 was conducted to determine the effects of stimulating hormone (FSH), luteinizing hormone (LH), prolactin (PRL) and growth hormone (GH) on the Aqp1 mRNA expression in Gc and Tc cells. Incubation medium was M199 medium (Sigma, St. Louis, MO, USA) containing nystatin (120 U/mL) (Sigma) and gentamicin (0.05 mg/mL), (Krka, Novo Mesto, Slovenia). Aliquots of Gc were initially cultured in: 1/12-well plates (Sarstedt, Equimed, Nümbrecht, Germany) 1.0 × 10^6^ cells per well and Tc 2.5 × 10^5^ cells per well without test compounds for 48–72 h to allow cell attachment to the wells (37 °C, 2% BSA, 10% FCS, 95% air/5% CO_2_). Following 48–72 h of attachment, cells were cultured with treatments (37 °C, 2% BSA, 5% FCS, 95% air/5% CO_2_) for the next 24 h, 1.0 mL fresh M199/FCS alone was added to the control cultures, while to the experimental cultures, FSH, LH, PRL and GH were added in a concentration of 100 ng/mL (Sigma). When the experiments were terminated, the adherent cells were washed with PBS and then harvested and stored (−80 °C) for mRNA expression analysis.

Experiment 2 was conducted to determine the effects of FSH, LH, PRL and GH on the AQP1 protein expression in Gc and Tc cells. Cultivations were conducted according to the procedure described above. Each treatment was conducted in four wells and each experiment was repeated three times. When the experiments were terminated, the cells (−80 °C) were collected and stored until all assays were completed for protein expression.

Experiment 3 was conducted to demonstrate the subcellular distribution of AQP1 protein in the Gc and Tc cells. Cells were isolated and cultured on Mini Cell slides (Merck Millipore, Burlington, MA, USA). Aliquots of Gc and Tc were 1.0 × 10^5^ cells per well/500 µL medium. The cells were cultured with treatments as described above (M199, 2% BSA, 5% FCS, 95% air/5% CO_2_) for the next 24 h. When the experiments were terminated, the cells were prepared for immunofluorescence.

Experiment 4 was conducted for co-culture experiments to demonstrate the effects of FSH, LH, PRL and GH on the AQP1 mRNA and protein expression in Gc and Tc cells. For co-culture experiments, viable Gc and Tc, were inoculated at a concentration of 2 × 10^6^ and 0.5 × 10^6^ cells/well, respectively, in tissue culture plates, which reflects typical ratios as observed in vivo, as described previously [61]. The media and incubation conditions with experimental factors are described as above. When the experiments were terminated, the cells (−80 °C) were collected and stored until all assays were completed for mRNA and protein expression.

Experiment 5 swelling assay was performed as described by [30]. At the beginning of the assay after incubation of Gc and Tc cells with hormones as already described, the medium was removed and fresh medium was added to the wells, after 15 min the cells were incubated with or without PMB 50 µM (Sigma Aldrich, St. Louis, MO, USA) and then exposed to a hypotonic medium (H_2_O: M199 3:4, osmotic pressure: 161 mosm) for 30 s. Photographs (×40) were taken from 0 to 30 s, at intervals of 3 s. and exposed to Image-Cell-F version 6.0 (Olympus, Japan) which was used to measure the volume of the cells (AnalySIS, Olympus, Japan), as described previously [63]. The diameters were measured and volumes calculated based on the assumption that the granulosa and theca cell approximates a complete sphere. The data are presented as the percent of initial volume.

### 4.3. RNA Extraction and Real-Time PCR

Total RNA was isolated from granulosa and theca cells with the “Total RNA” kit (A&A Biotechnology, Gdynia, Poland) following the manufacturer’s recommendations and quantified spectrophotometrically. The integrity of the product was confirmed on 1.5% agarose gel. Reverse transcription (RT) was performed using an Enhanced Avian HS RT-PCR Kit (Sigma Aldrich) and a mix of dNTPs and random hexamers as primers. The RT product was kept frozen at −20 °C for PCR analysis. Quantitative Real-Time PCR was used to establish dynamic changes in *Aqp1* mRNA expression. The following primers sequences were used [40,41]: *Aqp1* forward CAGCGAGTTCAAGAAGAAG, *Aqp1* reverse GCGACACCTTCACGTTATC, *GAPDH* forward GACCTCCACTACATGGTCTA, *GAPDH* reverse AAGATGGTGATGGCCTTTC, *PPIA* forward GCACTGGTGGCAAGTCCAT and *PPIA* reverse AGGACCCGTATGCTTCAGGA (access No.: AY266299) available in GeneBank, this study. Glyceraldehyde 3-phosphate dehydrogenase (*GAPDH*) and Cyclophylin A (*PPIA*) were used as normalization controls. Real-Time PCR was performed (7300 Real-Time PCR system; Applied Biosystems, Foster City, CA, USA) as describe previously [40]. Each experiment was independently repeated at least three times and the fold change in the expression of each gene was analyzed via the 2^−ΔΔ*C*t^ method.

### 4.4. SDS-PAGE and Western Blot

GCs and Tc were harvested, rinsed twice with PBS, lysed in denaturing lysis buffer (RIPA) with protease inhibitors on ice for 30 min and then centrifuged (12,000× *g*) for 15 min at 4 °C. Protein concentration was determined by the Bradford method. Western blot analysis was performed as described previously by Skowronska et al. [31].

### 4.5. Immunofluorescence for AQP1 in Gc and Tc Cultures

Immunofluorescence was performed as previously described [35]. Briefly, cultures were fixed in 4% paraformaldehyde, rinsed with phosphate-buffered saline (PBS), permeabilized with 0.2% saponin 0.01 M PBS for 10 min and incubated for 30 min at 37 °C in PBS containing 10% normal goat serum (NGS). The slides were then incubated overnight at 4 °C with an anti-AQP1 antibody (1:200). The AQP1 antibody was previously characterized, respectively by Terris et al. [64]. After washing, coverslips were incubated with Alexa Fluor 488 donkey anti-rabbit IgG conjugated secondary antibodies for one hour. Nuclei were stained with TO-PRO^®^-3 (Invitrogen, Carlsbad, CA, USA). The slides were then mounted with Fluorescence Mounting Medium (DAKO). Fluorescence localization was detected by fluorescent microscopy (Olympus, Japan).

### 4.6. Statistical Analysis

The data were analyzed by Statistica software (StatSoft Inc., Tulsa, OK, USA). The effect of the treatment was performed by a one-way analysis of variance for repeated measurements followed by the NIR Fisher post-hoc test. Statistical significances were assigned at *p* ≤ 0.05 while not significant differences indicate *p* > 0.05. The data are presented as means ± S.E.M.

## Supplementary Materials

Supplementary materials can be found at www.mdpi.com/1422-0067/19/1/5/s1.

## Abbreviations

AQPs	Aquaporins
FSH	Follicle-stimulating hormone
LH	Luteinising hormone
PRL	Prolactin
GH	Growth hormone
IGF-S	Insulin-like growth factors
Gc	Granulosa cells
Tc	Theca cells
GAPDH	Glyceraldehyde 3-phosphate dehydrogenase
PPIA	Cyclophylin A
