# Supplementary material for: Pituitary Gonadotropins, Prolactin and Growth Hormone Differentially Regulate AQP1 Expression in the Porcine Ovarian Follicular Cells
Source: Int J Mol Sci. 2017 Dec 21;19(1):5. doi: 10.3390/ijms19010005 (PMC5795957; doi:10.3390/ijms19010005)
Supplement: Supplementary file 1 [file ijms-19-00005-s001.pdf]

Table S1. Time courses of the volume change in the granulosa and theca cells of large porcine ovarian follicles.

Factors	Large follicles Granulosa cells area (µm^2)	Large follicles Theca cells area (µm^2)
-PMB	4603 ± 197	4668 ± 197
+PMB	3645 ± 107	3634 ± 106
+FSH	4673 ± 151	n/a
+FSH PMB	3731 ± 101	n/a
+LH	n/a	4089 ± 88
+LH PMB	n/a	3773 ± 131
+PRL	4596 ± 176	4153 ± 152
+PRL PMB	3945 ± 221	3971 ± 177
+GH	4416 ± 111	3936 ± 112
+GH PMB	3942 ± 116	3763 ± 103

Table S2. Time courses of the volume change in the granulosa and theca cells of medium porcine ovarian follicles.

Factors	Medium follicles Granulosa cells area (µm^2)	Medium follicles Theca cells area (µm^2)
-PMB	4413 ± 197	4534 ± 182
+PMB	3721 ± 112	3669 ± 115
+FSH	4569 ± 169	n/a
+FSH PMB	3894 ± 158	n/a
+LH	n/a	4292 ± 117
+LH PMB	n/a	3842 ± 114
+PRL	4702 ± 177	4150 ± 129
+PRL PMB	4014 ± 189	3849 ± 147
+GH	4537 ± 109	3994 ± 104
+GH PMB	3859 ± 105	3732 ± 101

Supplementary Data

Time courses of the volume change after the hypotonic stimulation (30 s) in the granulosa and theca cells of medium and large porcine ovarian follicles exposed to the examined factors with (+PMB) or without (-PMB) the presence of AQPs blocker. Data are mean ± S.E.M of five separated measurements of five separated experiments performed on different days. n/a: not applicable.
